# Extra-Limites

**Published:** 1837-01-01

**Authors:** 


### EXTRA-LIMITES.

### RECLAMATION OF DR. HAMILTON.

Dr. Hamilton, the Professor of Midwifery in the University of Edinburgh, has published, and widely circulated, a long and somewhat discourteous Letter, addressed to Dr. Johnson, complaining of some observations which we have made on his recent work in the number of this Journal for last July, and charging us with misrepresentation of his opinions and perversion of recorded facts.

We have no doubt that we shall be able to satisfy most of our readers, and probably Dr. Hamilton himself, that he has acted hastily and imprudently in challenging us to a re-examination of some parts of his work.

Many of the statements in it are so unguarded, that we were quite surprised, at the time, that any experienced practitioner could have published them without reserve or qualification. To accuse a reviewer of dishonesty and "mala-fides," is a very grave and serious charge; but one, which we unhesitatingly reject, as applicable to ourselves. It is not by such practices that the Medico-Chirurgical Review has obtained that confidence of the profession, and that influence on medical opinions, which Dr. Hamilton must have felt that it has. We proceed at once to examine the objections which he has made.

Dr. Hamilton's first charge of unfairness is founded on the following passage in our review: "Dr. H. asserts that he is able to distinguish unequivocally ovarian from ascitic dropsy, after paracentesis, by the following signs—'the peculiar appearance of the fluid, which in dropsy of the ovarium is commonly amber-coloured, and of the consistence of melted calf's-foot jelly, but more particularly the collapsed sac, distinctly perceivable on the day after tapping, like the contracted uterus on the day after delivery, afford certain evidence of dropsy of the ovarium.' On other subjects, as well as upon this, our author's language is far too peremptory, and, if unreservedly believed, might mislead an inexperienced practitioner." P. 41.

Dr. Hamilton's answer to these remarks is thus expressed:—

"The reviewer has called in question a fact which is so well known in this city, that it must excite surprise here that any practitioner should be ignorant of it, viz. that the most certain method of distinguishing dropsy of the ovarium from ascites is by tapping. In the former disease, the fluid drawn off is ropy and gelatinous, and after the operation, the collapsed sac can be as plainly distinguished through the parietes of the abdomen, as the collapsed uterus after child-bearing." 4.

Dr. Hamilton is quite in error, when he asserts that we have denied that "the most certain method of distinguishing dropsy of the ovarium from ascites is by tapping." We have never done so. We abide by the language, which we made use of at first—that neither he, nor any physician, is able to distinguish unequivocally ovarian from ascitic dropsy by any of the signs which he has mentioned.

Dr. Hamilton is surely well aware, that the fluid of ovarian dropsy is not uniformly "ropy and gelatinous;" but that sometimes, especially when there is only one large unilocular sac present, it is a thin, limpid, and straw-coloured serum. We may also add that the fluid in ascites is occasionally, as when the effusion is connected with disease of the peritoneum, of an almost syrupy consistence, and is at the same time turbid, and much discoloured.

With respect to the assertion that in all cases "the collapsed sac can be as plainly distinguished through the parietes of the abdomen, as the collapsed uterus after child-bearing," Dr. Hamilton must draw very largely on the credulity of his professional brethren, if he expects an unreserved assent to such a doctrine. Every pathologist knows that the sac or cyst of ovarian dropsy is sometimes quite thin and membranous, adhering, it may be, to the surrounding viscera, and unattended with any enlargement of the proper substance of the ovary. It is utterly impossible that such a sac, when emptied of its contents by tapping, can be as plainly distinguished through the abdominal parietes as the contracted uterus after delivery. Our author no doubt meaned to state that in many cases the collapsed sac is to be felt; certainly not in all, as he incautiously leads the reader to suppose.

Dr. Hamilton's second and third accusations are directed against some remarks, which we have made on a statement in his work that, "there are two circumstances which invariably attend pregnancy during the early months, viz. suppression of the catamenia, and a perceptible change on the surface of the mammæ surrounding the nipples." We objected to the authoritative and unqualified manner in which these assertions are made, and we expressed our sentiments thus:—"Dr. H. admits that, in some pregnant women, there may be periodical bloody discharges from the vagina; but he qualifies this admission by stating that there is always an irregularity in the date of the recurrence, (though, perhaps, to the extent only of a day or two,) as well as in the duration, the quantity and the quality of these discharges.* He may well add, 'in all doubtful cases accurate information on these points ought to be ascertained;'—the very difficulty consists in obtaining such accurate information. Let it not, however, be supposed that we differ in opinion from the Professor on the point at issue; our well-assured conviction is, that the *genuine* menstrual flow does not, and cannot take place during pregnancy; but of this we are equally certain, that not one woman in a thousand can distinguish *it* from other sanguineous discharges; and it is on their testimony that the accoucheur must in general found his opinion."

Dr. Hamilton replies:—

"In the 137th page of my work, there is the following strong denial of my belief in the possibility of '*periodical* bloody discharges during pregnancy.'

That, in some individuals a flow of blood is directed to the uterine arteries, during the first months of pregnancy, exactly at the regular periods of menstruation, indicated by a bloody discharge per vaginam, has been often alleged, but it does not consist with the experience of the author, that there ever was, strictly speaking, such an occurrence."

Not satisfied with having suppressed this passage, the reviewer has thought fit to substitute a different word for that which I had used, and in making this substitution, he has selected a word which not only misrepresents my opinion, but also renders the sentence ludicrous. How can periodical bloody discharges take place, if 'there be an irregularity in the date of the recurrence?' The reviewer has substituted *periodical* for IRREGULAR, which is my expression." 7.

This answer, it will be observed, is founded on a verbal quibble about the exact meaning of the word "periodical." Dr. Hamilton does not deny that he has admitted, in another part of his work, that, in some pregnant women, there are bloody discharges recurring about the same period of every month, and that the period of this monthly recurrence may not vary more than a day or two; but he intrenches himself against the objections which we have made, by stating that these bloody discharges do not return "exactly at the regular periods of menstruation." We may ask, do the catamenia always return exactly at the same period? Dr. Hamilton may be pleased to split a hair for the sake of dispute: we have no inclination to imitate him. His opinion is at variance with almost every authority, Denman excepted, on the subject. We could cite a score or two, if it was necessary; but shall adduce only one, whose experience and talents will be acknowledged by all. Dr Blundell states, in his Principles and Practice of Obstetricy, published two years ago:—"Notwithstanding what Denman has said to the contrary, I have myself known women in whom, during

* Dr. Hamilton, in extracting this passage from our Review, very unfairly halves it in two, and gives the reader only the former half, leaving out altogether those important words, "though, perhaps, to the extent only of a day or two."

the first three or four months, the catamenia have continued to flow, though not in so large a quantity, nor so long, as if they were not pregnant." 165.

As to the other "invariable sign" and "unequivocal mark" of pregnancy, the perceptible change on the surface of the mammæ surrounding the nipples—we remarked at p. 43 of our Review—"Our own experience, and, we may add, that of almost all the latest and best writers on midwifery and on medical jurisprudence, do not certainly warrant that unqualified and unequivocal reliance on the changes of the areolæ, as a sign of early pregnancy, which Dr. H. puts in them."

Dr. Hamilton replies in the following terms :

"With every respect for writers on Medical Jurisprudence, I certainly cannot bow to their authority on a practical point of this nature, and so far from 'all the latest writers on Midwifery' disregarding this test, one of the latest and best writers, Dr. Montgomery of Dublin, has described this change round the nipple with a degree of minuteness which could only be suggested by truth, and has expressed himself in regard to the value of this test, in as strong language as I could have done." 8.

He proceeds to give a long extract from Dr. Montgomery's essay, in support of his opinion respecting the turgescence of the areolæ, but very wisely refrains from citing any other authority. We may, 'in limine,' ask Dr. Hamilton, if it is fair and honourable in him to substitute the expression, "all the latest writers on midwifery," for that of "almost all the latest and best writers on midwifery"? We were well aware of Dr. Montgomery's opinions, and have alluded to them in our review of his essay, two years and a half ago ; but at the same time we know, and Dr. Hamilton knows, that by far the greater number of obstetrical writers and obstetrical observers do not admit that the turgescence of the areolæ is, as he maintains, an invariable and unequivocal sign of pregnancy. It is almost needless to refer to authorities on this point—Denman, Gooch, Burns, Velpeau, and a host of others, are opposed to our author. Even Dr. Montgomery himself, whose authority Dr. Hamilton so gladly calls to his support, but who is a much more cautious writer than Dr. H., thus expresses himself at one part of his essay :—

"At the same time, it should be observed that the areola does not always, in pregnant women, present all the characters we have described as belonging to it. We have seen it at the time of labour presenting the dark circle alone, without the prominence of the glandular follicles:" and again; "Their absence ought not to decide our opinion against the existence of pregancy, though their presence would be with us a very convincing proof of previous conception."

So much for the second and third charges of unfairness and misrepresentation brought against us by our honourable opponent.

The fourth charge need not detain us, as Dr. Hamilton is fortunately not disposed on this occasion to accuse us of any unfairness, or wish to misrepresent his statements. The only difference betwixt him and ourselves respects the treatment of the rare case, in which the first stage of labour is protracted in consequence of a rigid and constricted state of the cervix uteri. Dr. H. recommends the accoucheur to "introduce his finger above the stricture, and to press from within outwards on the resisting band of the uterus during each successive pain." In reviewing this passage, we observed that—

"The Professor's advice must be acted upon with great circumspection and gentleness. We are great enemies to all unnecessary manual interference in the management of labour, and we should be rather unwilling to carry the finger within the cervix uteri, above the seat of the stricture, for the purpose of pressing upon it from within." "We prefer the method recommended by Dr. Burns, of making the pressure merely on the anterior edge of the os uteri, as already explained in an extract given above." 14.

We see no reason to alter our opinion, and we are confident that most accoucheurs will agree in the propriety of our suggestions.

The fifth charge is introduced in the following manner :

"The next subject in which I must express my strong sense of the want of candour of the reviewer, is in the following words, p. 48.—'We wish that we could with propriety say as much for the succeeding chapter on the management of the second stage. Thirty pages of print to be occupied with recommending the accoucheur to apply his naked (without a napkin) hand to the perinæum, when the head is pressing down, and to smear the parts with soft lard. In truth, this is the sum and substance of the whole chapter.'

Perhaps it would be difficult to find in the annals of medical criticism, a more uncandid and unfounded assertion than that contained in this sentence." 15.

Dr. H. proceeds to vindicate himself by stating, that he deemed it necessary to quote the directions of Dr. Denman, of Dr. Davis, of Professor Burns, of MM. Baudelocque and Velpeau, for the purpose of shewing that the practice of all those gentlemen was both erroneous and dangerous; and moreover, that he has given other directions for the management of the second stage of labour, which we have neglected to mention—namely, "to remain by the patient from the time that the head of the infant clears the os uteri, and to force the perineum towards the pubes during every pain after the head begins to protrude."

To judge of the propriety of our strictures, the reader will find it necessary to examine the passage, which seems to have given the Professor so much annoyance, in connexion with what goes before. We had mentioned favourably the chapter on the management of the first stage of labour, and we gave the author credit for some valuable practical suggestions. The two next chapters appeared to us most uselessly prolix, more especially in a work, which so far from professing to give a systematic account of midwifery, has for its express object "to record," says the author, "my own opinions and discoveries."

The directions, with the exception of the last, are all very good, and we have not found any fault with them, but only with the excessively tedious manner, in which they are announced. In truth, the practice of every prudent accoucheur must be nearly the same in managing the second stage of natural labour, from the time that the head begins to protrude on the perineum until it is expelled. As a matter of course, he will not leave the bedside of his patient; and his great and leading duty is to give a broad and uniform support with his hand to the perineum, when the head presses down during the labour-pains. As for the use of lard, it is in many cases quite unnecessary, the parts being sufficiently lubricated by the vaginal discharges. However, it can do no harm; but then this, as well as the other directions, might have surely been expounded in less space, than thirty pages of print. The prolix and wearisome review of other writers, with extracts from their works, was most unnecessary in a book whose "professed object was to record the opinions and discoveries" of an author who, on certain occasions, as we shall presently see, does not deign even to allude to the opinions and discoveries of others, on a subject of the very greatest interest.

When the Practical Observations reach a second edition, Dr. Hamilton's good sense will, no doubt, lead him to compress the thirty wearisome pages into the compass of two or three.

We have now come to the last accusation which Dr. Hamilton prefers against us, and as it involves a question of great physiological interest, we shall treat of it at rather greater length, not certainly in the angry mood of our opponent, but with the calm and dispassionate temper of a searcher after truth.

Dr. H. charges us with "mala fides,"—in plain English, with wilful falsehood—for having penned the following passage at page 50, of our Review:

"Dr. H. says, that in his long experience he has in no instance found the pulse of the child before it begins to breathe, to exceed sixty beats in a minute. Of the truth of this he has repeatedly convinced himself by counting the pulsations of the cord, not only while the child was still in utero, but also after its expulsion before respiration commenced. The experience, however, of his friend and coadjutor Dr. Moir, and, we need scarcely add, of all other accoucheurs, is directly opposed to the Professor's statements."

On this passage Dr. Hamilton angrily comments.

"The assertion of the reviewer, that the experience of Dr. John Moir is directly opposed to the Professor's statements, is a perversion of a recorded fact, which I shall not stoop to characterize. Dr. Moir, in reporting his experiments with the stethoscope, has thus expressed himself, page 312:—'This effect of the contractions of the uterus, acting indirectly on the circulation of the fœtus through the medium of the brain, may account *for the fact, that the pulsations of the heart are only about* 60 *in infants who do not breathe on birth,* but in whom the circulation still goes on through the chord.'

By this quotation, it is evident that Dr. Moir bears testimony to the truth of my account of the action of the fœtal heart before breathing; while your reviewer avers that the experience of Dr. Moir is directly *opposed* to the Professor's statements."

The reader, who has not referred to our Review, might no doubt suppose, from the extract which the Professor has so cunningly selected, that we had dishonestly concealed or perverted the results of Dr. Moir's experience, for the express purpose of contradicting our author's statements. But what is the truth? The very next passage to the one which Dr. Hamilton has quoted runs thus:—

"In several cases, where turning was necessary, Dr. Moir found that the pulsations of the fœtus in utero were 120 and upwards, and moreover that the number of pulsations ascertained with the hand corresponded exactly with those which he discovered by means of the stethoscope. It deserves, however, to be mentioned, that Dr. Moir invariably found that the number of the fœtal pulsations diminished greatly (sometimes from 120 to between 60 and 70), whenever uterine contraction supervened, and again increased, whenever the contraction was over. This circumstance is the more worthy of being noticed, as Dr. E. Kennedy has not alluded to it in his excellent work, and the reverse of it is maintained by Dr. Hohl, whose work we reviewed in the Foreign Periscope of our last number but one.

The assertion of Dr. Hamilton, that the pulse of the child immediately after delivery, provided respiration has not begun, is generally sixty or thereabouts, is surely quite incorrect."

We are called upon, out of respect to our character, to charge Dr. Hamilton with most disingenuous dealing, for having thus wilfully clipped and detached portions of our Review, in the vain hope of detecting us in dishonesty.

Will any one deny after perusing the foregoing extracts (or, if he prefers it, after reading Dr. Hamilton's book), that the experience of Dr. Moir is *directly opposed* to the Professor's unqualified and unguarded assertion, that the pulse of the fœtus is generally sixty or thereabouts? Dr. Moir seems to be too prudent a man to commit himself in a notorious error, to please Dr. Hamilton or any other person; and we find therefore that, in the letter which he has written to Dr. H. and which is printed in the pamphlet, he very properly repeats his former statement, that it is only under particular circumstances, that the pulse of the child is so low as 60 or 70.

We gave Dr. Moir credit for having pointed out to obstetricians the changes on the fœtal pulse during uterine contractions, and we invited the attention of practitioners to this hitherto unobserved occurrence. He was no doubt too well acquainted with the experience of Mayor, Laennec, Kergaradec, Velpeau, Ferguson, Kennedy, &c. to hazard the bold assertion, that the pulse of the fœtus is usually sixty or thereabouts. Not so with Dr. Hamilton. We shrewdly suspect that he values very cheaply the opinions and experience of other practitioners; perhaps he does not take the trouble of informing himself of them. He is candid enough to confess that he does not read our Review very regularly, and it seems that he was not aware of our censures, for nearly four months after they appeared.

Perhaps, therefore, he has not heard of the work of Dr. Hohl on Obstetrical Auscultation, of which we gave a minute analysis in the number for last January, and which we recommend to the Professor's notice. We need not mention the names of other authors—for they are better known—all of whom most unhesitatingly agree that the pulse of the fœtus is usually very rapid, beating from 120 to 140 or upwards.

In taking leave of Dr. Hamilton, we have much pleasure in repeating our expression of respect and admiration for his talents and great practical skill. Without these, he never could have attained the high rank which he has so long held as a skilful accoucheur, and as an able professor.

